# The European pharmacy market: the density and its influencing factors

**DOI:** 10.1093/eurpub/ckac131.027

**Published:** 2022-10-25

**Authors:** S Schwaabe

**Affiliations:** Chair of International Relations, School of Social Science and Technology, Technical University Munich, Munich, Germany

## Abstract

Community pharmacies deliver high-quality health care and are responsible for medication safety. During the pandemic, accessibility to the nearest pharmacy became more essential to get vaccinated against Covid-19 and to get medical aid. The government’s goal is to ensure nationwide, reachable, and affordable medical health care services by pharmacies. Therefore, the density of community pharmacies matters. Overall, the density of community pharmacies is fluctuating, with slightly decreasing tendencies in some countries. The research question is: upon which conditions depends the variance in the density of community pharmacies in Europe? So far, the literature has shown that changes in the system affect prices and density. However, a European overview of the development of the density of community pharmacies and its triggers is still missing. This research is essential to counteract against decreasing density consulting in a lack of professional health care through pharmacies. I focus on liberal versus regulated market structures, mail-order prescription drug regulation, and third-party ownership consequences. In a panel analysis, the relative influence of the measures is examined across 27 European countries over the last 21 years. The results show that regulated pharmacy markets have over 10.75 pharmacies/100.000 inhabitants more than liberal markets. Further, mail-order prescription drugs decrease the density by -17.98 pharmacies/100.000 inhabitants. Countries allowing third-party ownership have 7.67 pharmacies/100.000 inhabitants more. The results are statistically significant at a 0.001 level. The output of my analysis recommends regulated pharmacy markets, with a ban on mail-order prescription drugs allowing third-party ownership to support nationwide medical health care through community pharmacies.

**Key messages:**

• Regulated pharmacy markets, with a ban on mail-order of prescription drugs and third-party-ownership have a positive effect on pharmacies density.

• The results of my analysis provide the government with measures to improve professional nationwide health care coverage for the population through pharmacies.

